# Divergent amyloid trajectories distinguish normal aging from Alzheimer's disease progression

**DOI:** 10.64898/2026.09.08.748727

**Published:** 2026-09-14

**Authors:** Mingzhao Tong, Tianchuan Gao, Yurika Upadhyaya, Kwangsik Nho, Shiaofen Fang, Andrew Saykin, Jingwen Yan, for the Alzheimer's Disease Neuroimaging Initiative

## Abstract

Aging is the greatest risk factor for Alzheimer's disease (AD), yet how and when AD-related pathological progression diverges from aging remains poorly understood. This distinction is particularly difficult at early stages, when clinically and biomarker-defined populations contain individuals following fundamentally different trajectories. Here, we model AD progression as a deviation from aging using longitudinal amyloid PET and a self-supervised trajectory-learning framework. In ADNI, the learned trajectory revealed a shared early path that bifurcated into an aging branch and an AD-related branch. The two branches showed distinct profiles in amyloid burden, cognitive decline, risk of progression to AD dementia, and genetic risk. Unseen participants from the independent NACC cohort were projected onto the trajectory without retraining, further reproducing key branch-specific biological and genetic patterns. Together, the bifurcating trajectory provides a biologically grounded framework for resolving early-stage heterogeneity and enabling risk stratification beyond binary amyloid status.

## Full Text Availability

The license terms selected by the author(s) for this preprint version do not permit archiving in PMC. The full text is available from the preprint server.

